# Fuzi alleviates cold-related rheumatoid arthritis via regulating gut microbiota and microbial bile acid metabolism

**DOI:** 10.1186/s13020-025-01123-z

**Published:** 2025-05-15

**Authors:** Juan Liu, Dandan Zhang, Yaochuan Zhou, Jinlu Wu, Wuwen Feng, Cheng Peng

**Affiliations:** 1 State Key Laboratory of Southwestern Chinese Medicine Resources, School of Pharmacy, Chengdu University of Traditional Chinese Medicine, Chengdu, 611137 China; 2 TCM Regulating Metabolic Diseases Key Laboratory of Sichuan Province, Hospital of Chengdu University of Traditional Chinese Medicine, Chengdu, 610032 China; 3 Key Laboratory of the Ministry of Education for Standardization of Chinese Medicine, Chengdu University of Traditional Chinese Medicine, Chengdu, 611137 China

**Keywords:** Fuzi, Rheumatoid arthritis, Gut microbiota, TGR5-cAMP-PKA signaling, NLRP3 inflammasome

## Abstract

**Background:**

Rheumatoid arthritis (RA) with cold pattern is an important type of RA according to the theory of traditional Chinese medicine. Fuzi (also known as the lateral roots of *Aconitum carmichaelii* Debx.) represents a typical traditional Chinese medicine that has been clinically used for treatment of the RA especially cold-related RA for thousands of years, yet its mechanism remains unknown.

**Purpose:**

The purpose of the research was to study the therapeutic effects of Fuzi on cold-related RA, and to investigate the mechanism of its action.

**Methods:**

Here, we investigated the pharmacological effects of Fuzi on cold-related RA using micro-CT, histopathological analysis, and inflammatory cytokine test. Then, a gut microbiota composition analysis in combination with fecal microbiota transplantation were used to confirm the role of gut microbiota in the therapeutic effects of Fuzi. Further, targeted bile acid metabolomics was used to screen the possible differential microbial bile acids involved in the mechanism of Fuzi. In vitro bioactivity analysis of differential bile acids was used to assess their anti-inflammation activity. Finally, western blot was used to investigate the signaling pathways of Fuzi in reducing the inflammation of cold-related RA.

**Results:**

The results showed that Fuzi alleviates cold-related RA by improving arthritis index, paw swelling, bone damage, and inflammatory cytokines. In addition, the ameliorative effect of Fuzi is dependent on gut microbiota such as the taxa Lachnospiraceae and Ruminococcaceae. Targeted analysis of fecal and serum bile acids showed that TCA and THDCA were the main differential metabolites. In vitro, TCA and THDCA showed anti-inflammation effects on RAW264.7 cells. Western blot showed that Fuzi regulates TGR5-cAMP-PKA signaling and NLRP3 inflammasome to reduce cold-related arthritis.

**Conclusion:**

Overall, our results demonstrated that Fuzi could regulate gut microbiota and microbial bile acid metabolism, the microbial metabolite THDCA acts on TGR5-cAMP-PKA signaling pathway and NLRP3 inflammasome to reduce cold-related arthritis. Our study suggests that supplementation of Fuzi or THDCA can be of great value for the prevention and clinical treatment of cold-related RA.

**Graphical Abstract:**

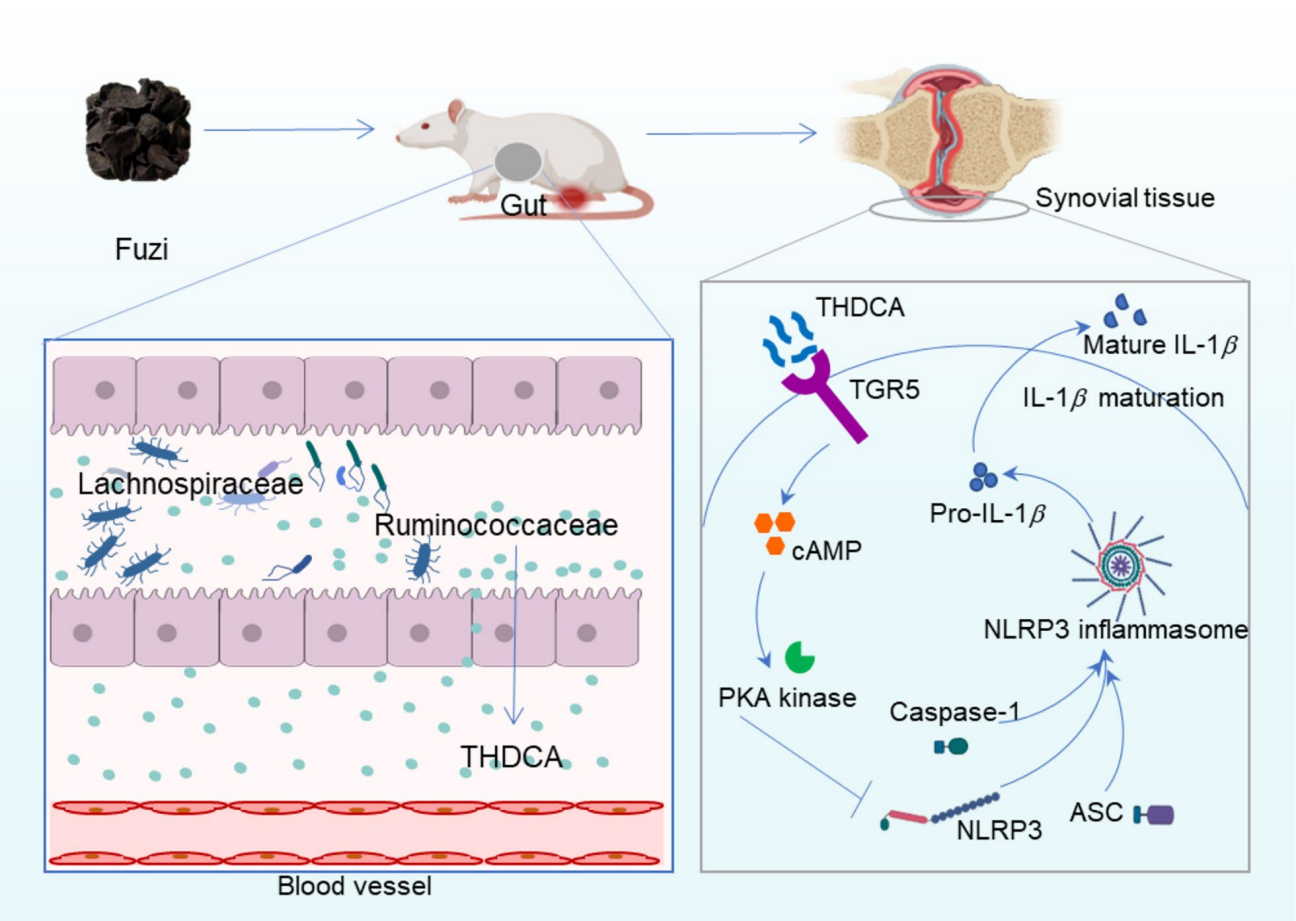

**Supplementary Information:**

The online version contains supplementary material available at 10.1186/s13020-025-01123-z.

## Background

Rheumatoid arthritis (RA) is a globally prevalent chronic and systemic immune disease that primarily targets the synovial joints, leading to persistent inflammation, pain, and progressive joint destruction. Affecting nearly 1% of the global population, RA poses a significant health burden, particularly among women and individuals aged 60–70 years [1, 2]. The exact cause of RA remains elusive, but it is understood to arise from a complex interplay among genetic predisposition, environmental factors, and dysregulation of immune system [3]. Current therapies for RA, including non-steroidal anti-inflammatory drugs, corticosteroids, anti-rheumatic drugs, biologics, etc., have been proved to be very effective in controlling symptoms and slowing disease progression [4]. However, these treatments often have significant drawbacks, such as adverse side effects, high costs, and the potential for diminished efficacy over time [5]. Moreover, not all patients respond adequately to existing therapies [6], highlighting the need for alternative treatment approaches that are both effective and offer a better safety profile. These limitations underscore the importance of exploring alternative therapies and novel mechanisms to enhance the management of RA.

As early as the classical Roman age, people in both Western and Eastern countries such as those in Greece and China hold the belief that arthritis was influenced by weather conditions such as cold, rain, and humidity [7]. Cold and wet weather conditions are generally thought to be bad for health, and warm and dry weather are thought to be good for people [8]. In traditional Chinese medicine theory, RA is generally classified into cold type and hot type [9]. The cold-related RA is characterized by the fearing of cold weather, joint severe pain in low temperature condition, and a white coating in the surface of the tongue [10]. According to traditional Chinese medicine theory, the cold weather, or sustained exposure to cold conditions, is one of the major causes of cold-related arthritis. In our previous study, we also demonstrated that prolonged cold exposure aggravates RA in the collagen-induced arthritis model [11]. Considering the importance and the representativeness of cold-related RA, it is imperative to find suitable alternative medicine to treat this type of disease.

Based on the theory of ancient hot and cold drug property, the traditional Chinese medicines that possess hot property have been extensively utilized in China and East Asia for treating cold-induced ailments and diseases [12]. As a typical hot traditional Chinese herbal drug, Fuzi (the lateral roots of *Aconitum carmichaelii* Debx.) is believed to expel cold, warm the meridians, and support Yang, thus improving circulation and reducing pain [13]. Due to its potent warming properties, it has been utilized for centuries in traditional Chinese medicine to treat RA in China [14]. Even though recent research has begun to explore the mechanisms behind Fuzi’s therapeutic effects on RA, its mechanism has not been fully studied, especially the cold-related RA. Gut microbiota has emerged as an important research direction in studying the mechanisms of herbal medicines. Our previous study found that in cold exposure induced hypothermia, Fuzi can regulate gut microbiota and bile acid metabolism to partly restore energy metabolism [15]. In another study, we found that cold exposure aggravates RA through modulating the gut microbiota and bile acid metabolism [11]. We therefore hypothesize that Fuzi can alleviate cold-related RA through regulation of gut microbiota and microbiota-related bile acid metabolism.

Herein, bovine collagen and isopycnic incomplete adjuvant were used to establish the RA model, and the RA rats were exposed to cold environment to establish the cold-related RA model. Gut microbiota composition analysis and fecal microbiota transplantation (FMT) were performed to validate the role of gut microbiota in treating RA by Fuzi. Targeted bile acid analysis was used to screen the key bile acid responsible for ameliorating RA. Further, the effects of key bile acid on RAW 264.7 cells and Western-blot was used to validate the role of bile acid in RA treatment by Fuzi. Our study suggests that Fuzi can potentially be applied as a regulator of the gut microbiota and bile acids to improve RA.

## Methods

### Preparation of Fuzi extract

Fuzi (Heishunpian) was obtained from Sichuan Jiangyou Zhongba Fuzi Keji Fazhan Co., Ltd. (Jiangyou, China). Fuzi pieces were weighted and then aqueously immersed with ten times volume of water for 30 min, and boiled in water for 5 h to eradicate the toxicity. Then, Fuzi was filtered, and an eightfold amount of water was supplemented and boiled for 3 h. The filtrate was mixed and concentrated to 0.625 g/ml for further chemical analysis and drug administration [15]. Then, it was chemically analyzed which we have previously reported elsewhere [16, 17].

### Animals, model induction, and drug administration

All the experiments were censored and approved under the supervision of Ethics Committee of the organization (CDUTCM). SPF-level adult male Wistar rats (280–300 g) were obtained from Chengdu Dasuo Experimental Animal Co., Ltd. (Chengdu, China). All rats were kept under a standard environment with the temperature at 22 ± 2 °C, the relative humidity at 60 ± 5%, a 12 h light/dark cycle, and with free access to water and food. The environment was controlled by a climate box DRXM-508 (Ningbo, China).

Animal experiment 1: After one week of adaptation, 30 rats were stochasticly divided into normal group (Normal) and collagen-induced arthritis at room temperature (CIA-RT) group, cold-related collagen-induced arthritis (CIA-cold) group, cold-related collagen-induced arthritis treated by methotrexate (CIA-cold-MTX) group, cold-related collagen-induced arthritis treated by Fuzi (CIA-cold-Fuzi) group. Normal and CIA-RT group were housed at 22 ± 2 °C conditions. CIA-cold, CIA-cold-MTX, CIA-cold-Fuzi rats were housed at 5 ± 2 °C conditions. The rats were immunized with 200 μL isopycnic incomplete Freund's adjuvant-emulsified bovine type II collagen (Chondrex, Inc., Redmond, WA, United States) via intradermal injection at the tail base. A further booster was performed with 100 μL emulsion prepared as initially used.

Animal experiment 2: After one week of adaptation, 24 rats were randomly divided into cold-related collagen-induced arthritis (CIA-cold) group, cold-related collagen-induced arthritis treated by Fuzi (CIA-cold-Fuzi) group, cold-related collagen-induced arthritis receiving microbiota from CIA-cold (Cold-Transpl.) group, and cold-related collagen-induced arthritis receiving microbiota from CIA-cold-Fuzi (Fuzi-Transpl.) group. All groups were housed at 5 ± 2 °C conditions, immunized on Day 0, and received booster immunization on day 7. CIA-cold-Fuzi group was treated with Fuzi and CIA-cold group were treated with normal saline from day 16 to the end of the experiment. The Cold-Transpl. group and Fuzi-Transpl. group were orally administered transplanted donor microbiota from CIA-cold and CIA-cold-Fuzi group, respectively. The fecal transplantation was performed every 2 days. The experimental design diagram for animal experiment 2 is shown in Fig. S1.

### Energy metabolism related hormone detection

The serum levels of triiodothyronine (T3), free triiodothyronine (FT3), thyroxine (T4), free thyroxine (FT4), thyrotropin-releasing hormone (TRH), and thyroid-stimulating hormone (TSH) were measured using ELISA kits (Cloud-Clone Corp., Wuhan, China) according to the manufacturer's instructions.

### Micro-CT analysis

Microcomputed tomography (micro-CT) was conducted on the excised ankle, tibia, and femur with the help of Quantum GX Micro-CT Imaging System (PerkinElmer, USA). The bones were scanned for 14 min at 90 kV and 80 μA, followed by the reconstruction of 3D images under the help of manufacturer's software.

### Histopathological analysis and ELISA

Knees were embedded in paraffin after fixation by 4% paraformaldehyde, and dehydrated for H&E staining using standard methods with a thickness of 3-μm. Serum interleukin (IL)-6, tumor necrosis factor-α (TNF-*α*), IL-1*β*, and interferon-*γ* (IFN-*γ*) were measured by ELISA kits (Multisciences Biotech, Co., Ltd., Hangzhou, China) according to the manufacturer's instructions.

### Gut microbiota analysis

The fresh feces were collected in sterile centrifuge tubes and quickly stored within − 80 °C condition. The 16S rRNA gene V3–V4 variable region was amplified using the upstream primer 338 forward primer 5’- ACTCCTACGGGAGGCAGCAG-3’; 806 reverse primer 5’-GGACTACHVGGGTWTCTAAT-3’. Specific method was described elsewhere [11].

### Bile acid analysis

A total of 10 mg of fecal matter was weighed and placed into a secure Eppendorf tube. About 25 mg of precooled zirconium oxide beads was added, followed by 200 μl of an acetonitrile/methanol (v/v = 8:2) mixture. The mixture was homogenized and then centrifuged at 13,500 rpm and 4 ºC for 20 min (Microfuge 20R, Beckman Coulter, Inc., Indianapolis, IN, USA). A 10 μl aliquot of the supernatant was transferred and diluted with 90 μl of an acetonitrile/methanol (v/v = 8:2) and ultrapure water mixture at a 1:1 ratio. After vortexing and centrifugation, the sample was prepared for injection. The injection volume was set at 5 μl. The detection parameters were previously described [11].

### Cell culture and cell viability assay

RAW264.7 cells were cultured in DMEM medium (Norbolide, Beijing, China). Cell incubator was set at 37 ºC with 5% carbon dioxide. RAW264.7 cells was seeded in 96-well plates at the concentration of 5 × 10^3^ cells/well and cultured for 24 h. Then, the medium was replaced by taurocholic acid (TCA) and taurohyodeoxycholic acid (THDCA) treatment at the concentration of 0, 1, 10, 50, 100, 200, 1000, 5000, 10,000 μM in culture medium. 10 μl of CCK-8 solution was added to each well in each group under light-protected conditions. The plates were then incubated for 3 h. The optical density at 450 nm was determined using the microplate reader, meanwhile the cell viability was calculated.

### Nitric oxide and nitric oxide synthase determination

Logarithmic phase RAW264.7 cells were seeded at 2 × 10^5^ cells/well. After 24 h of culture, the supernatant was discarded. The blank group was treated with complete culture medium; the LPS model group was treated with complete culture medium containing LPS (100 ng/ml); the TCA group was treated with complete culture medium containing LPS (100 ng/ml) and TCA (10, 50, 100 μM); the THDCA group was treated with complete culture medium containing LPS (100 ng/ml) and THDCA (10, 50, 100 μM). The plates were then incubated for 24 h. NO and iNOS level were determined according to the manufacturer's instructions.

### qRT-PCR

The total RNA from each cell sample was reversed into cDNA using synthesis premix and a T100 PCR instrument. The mRNA expression levels of inflammatory cytokines were determined using a fluorescence-based quantitative PCR kit. The relative quantification of mRNA was performed using the 2_T_^−ΔΔC^ method. The primers for GAPDH were GTGGAGTCCACTGGCGTCTT (Forward) and GTGCAGGAGGCATTGCTGAT (Reverse), and the primers for iNOS were CAGCTGGGCTGTACAAACCTT (Forward) and CATTGGAAGTGAAGCGTTTCG (Reverse). All primers used for qRT-PCR were designed using the Prime-BLAST platform (NCBI) and synthesized by Qingke Biotechnology (Beijing, China).

### Western blot

The synovial tissues were lysed with RIPA buffer. Denatured total protein was then loaded onto 10% or 12% gels to perform sodium dodecyl sulfate–polyacrylamide gel electrophoresis. The membranes were blocked and incubated with antibodies: anti-TGR5 (Novus Biologicals, USA), anti-Caspase-1 (Novus Biologicals), anti-cAMP protein kinase catalytic subunit (Abcam, UK), anti-NLRP3 (Abcam), anti-ASC (Abcam), anti-IL-1*β* (Abcam), anti-p-PKA substrate (Cell Signaling Technology, Danvers, MA, USA), anti-PKA substrate (LifeSpan Bioscience, Seattle, WA, USA), and anti-GAPDH (Affinity Biosciences, Jiangsu, China), followed by incubation with the anti-rabbit IgG (Aibixin, China).

### Statistical analysis

Shapiro–Wilk test was performed for checking normal distribution. Unpaired two-tailed Student's t-test and one-way ANOVA with Dunnett's multiple comparison test were performed to for normal distribution data. For not normally distributed data, Mann–Whitney U test and the Kruskal–Wallis test were used. Two-way ANOVA with Sidak's multiple comparison was used for data with more than two groups and two variables.

## Results

### Fuzi alleviated cold-related rheumatoid arthritis

**Fig. 1:**
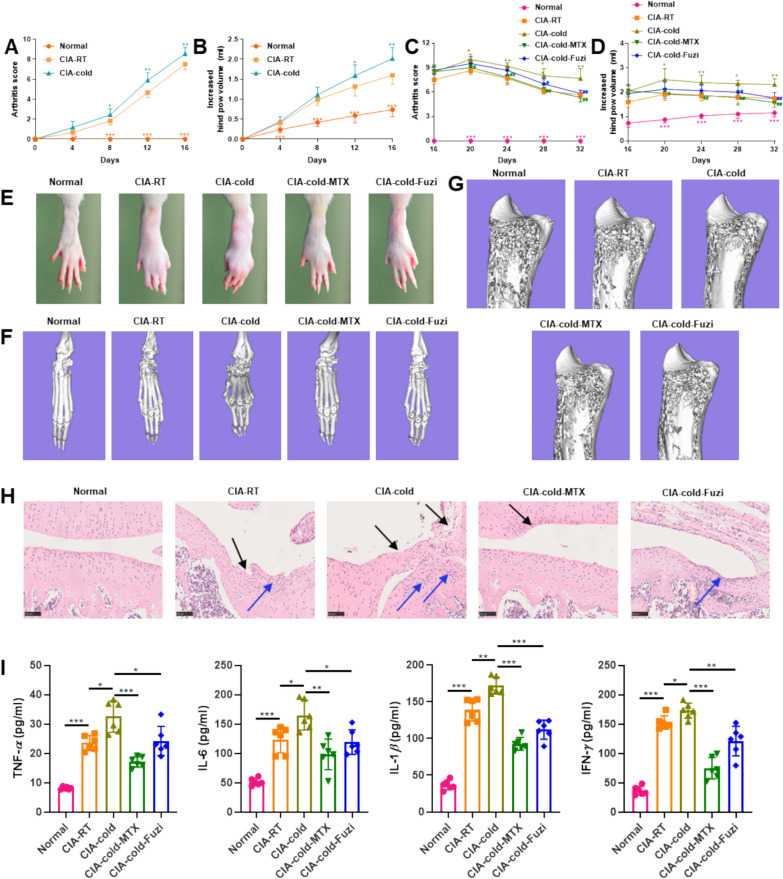
Effects of Fuzi on CIA-cold rats. **A **, **B** Arthritis scores (**A**) and paw swelling (**B**) of rats after 16 days of cold exposure (compared to CIA-RT). **C **, **D** Arthritis scores (**C**) and paw swelling (**D**) of rats after Fuzi treatment (Magenta and khaki ^*^, compared to CIA-RT group; green and blue ^#^, compared to the CIA-cold group). **E** Images of the rat hind paws. **F** Micro-CT pictures of the rat ankle joint. **G** Micro-CT images of the rat proximal tibia. **H** H&E-staining of knee joints (× 200). **I** Serum levels of inflammatory cytokines, including TNF-*α*, IL-1*β*, IL-6, and IFN-*γ*. Normal: Normal group; CIA-RT: Collagen-induced arthritis group; CIA-cold: Collagen-induced arthritis cold model group; CIA-cold-MTX: Collagen-induced arthritis cold model + methotrexate treatment group; CIA-cold-Fuzi: Collagen-induced arthritis cold model + Fuzi treatment group; MTX: Methotrexate; Fuzi: Fuzi decoction. Data are presented as mean ± standard deviation (mean ± *SD*)

At the end of the 16-day cold exposure, the arthritis index and paw swelling in CIA rats were significantly increased compared to the normal group (*p* < 0.001) (Fig. 1A , B). Compared to CIA rats, the arthritis scores and paw swelling in CIA-cold rats increased significantly further after 16 days of cold exposure (*p* < 0.01) (Fig. 1A , B). Importantly, after 16 days of treatment with methotrexate and Fuzi (day 32), the arthritis index and paw swelling in CIA-cold rats remained significantly higher than those in the CIA group, but the arthritis index and paw swelling in the CIA-cold rats treated with methotrexate and Fuzi (CIA-cold-MTX and CIA-cold-Fuzi groups) were significantly lower than those in the CIA-cold group (Fig. 1C , D). In addition, CIA-cold rats showed reduced energy metabolism-associated hormones compared with CIA and normal rats, while Fuzi significantly increased these hormones (Fig. S2A − F). These results suggest that Fuzi effectively improved the arthritis index, paw swelling, and energy metabolism in CIA-cold rats.

**Fig. 2:**
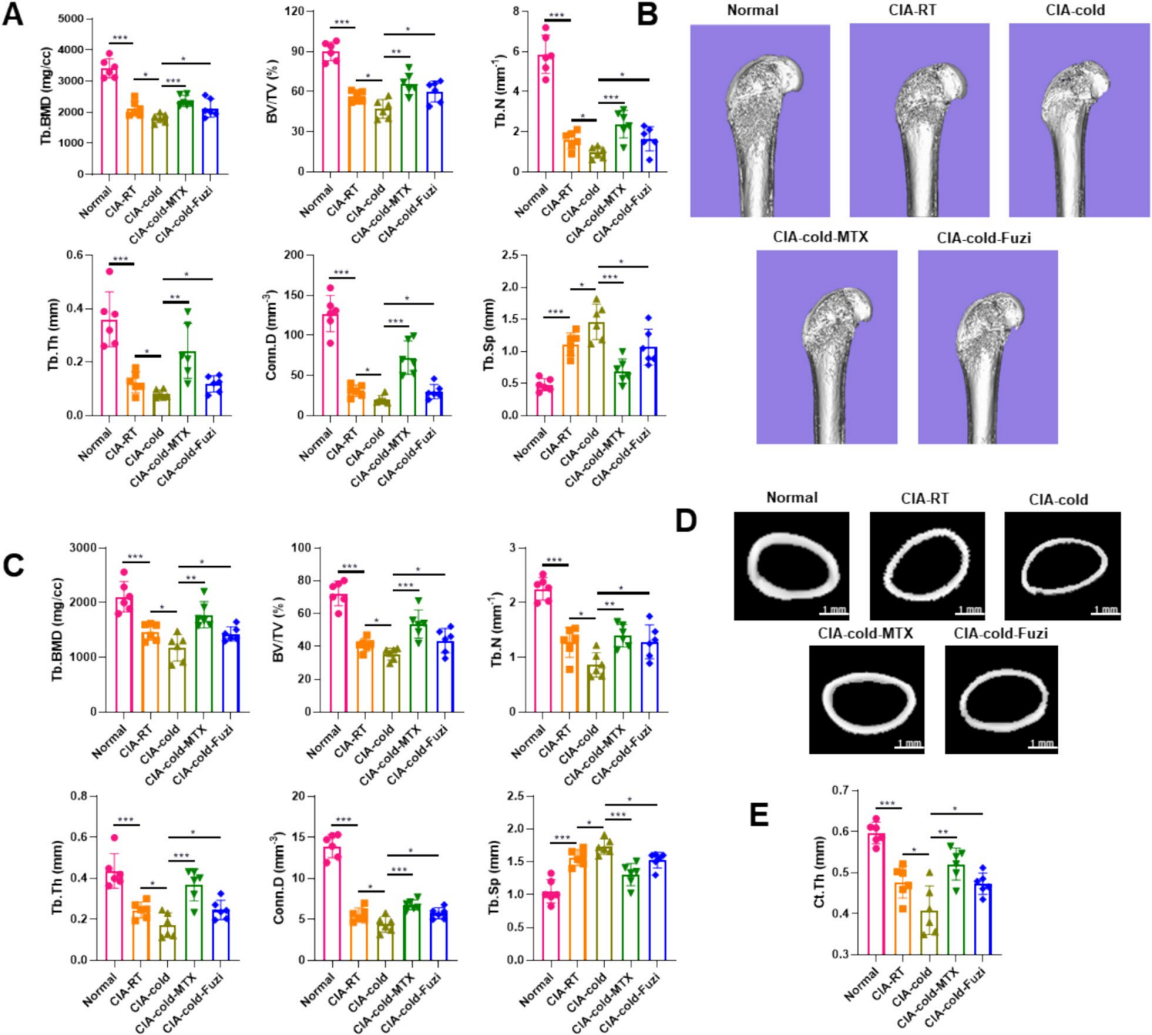
Effects of Fuzi on the severity of arthritis in CIA-cold rats. **A** Trabecular bone parameters of the tibia. **B** Micro-CT images of the distal femur. **C** Trabecular bone parameters of the femur. **D** Micromicro-CT images of the midshaft of the femur. **E** Cortical bone thickness (Ct.Th) of the midshaft femur

Photographs of the hind limbs of the rats showed that CIA rats exhibited ankle joint swelling and deformity, with the severity of ankle joint swelling and deformity being more pronounced in CIA-cold rats than that in CIA rats. After treatment with methotrexate and Fuzi, the degree of ankle joint swelling and deformity was alleviated (Fig. 1E). Micro-CT scans and 3D reconstruction of the joints showed that the extent of ankle joint deformation and erosion was improved after treatment with methotrexate and Fuzi (Fig. 1F). Furthermore, compared to CIA-cold rats, the trabecular bone parameters of the tibia and femur were significantly enhanced in the CIA-cold-MTX group and CIA-cold-Fuzi group (Figs. 1G and 2A –E). These parameters include mineral density of the bone (BMD), volume fraction of the bone (BV/TV), the number of trabecular (Tb.N), the thickness of trabecular (Tb.Th), the density of tge trabecular connectivity (Conn.D), and the thickness of cortical (Ct.Th) of the femoral midshaft. On the contrary, the trabecular separation (Tb.Sp) of the tibia and femur was significantly reduced (Figs. 1G and 2A –E). These results indicate that Fuzi can alleviate bone damage in cold-related RA rats by reducing ankle joint swelling and deformity and increasing trabecular bone in the tibia and femur.

Histopathological sections of knee joint tissue revealed multiple areas of cartilage loss (black arrows) and lymphocyte infiltration (blue arrows) in the knee joints of CIA-cold rats (Fig. 1H). After treatment with methotrexate and Fuzi, the cartilage loss (black arrows) and lymphocyte infiltration (blue arrows) in the knee joint tissue were improved. These findings suggest that Fuzi can improve knee joint inflammation in CIA-cold rats by reducing cartilage loss and lymphocyte infiltration in the knee joint tissue. Consistent with our previous CIA-cold studies, the levels of TNF-*α*, IL-1*β*, IL-6, and IFN-*γ* in CIA rats were significantly higher than those in normal rats, and the levels of serum inflammatory cytokines were significantly increased in CIA-cold rats compared to CIA rats (Fig. 1I). Methotrexate and Fuzi intervention significantly reduced the levels of serum inflammatory cytokines in CIA-cold rats (Fig. 1I). In summary, the severity of arthritis in CIA-cold rats is consistent with our previous studies, and Fuzi can effectively alleviate the severity of arthritis in CIA-cold rats.

### Ameliorative effect of Fuzi is dependent on gut microbiota

**Fig. 3:**
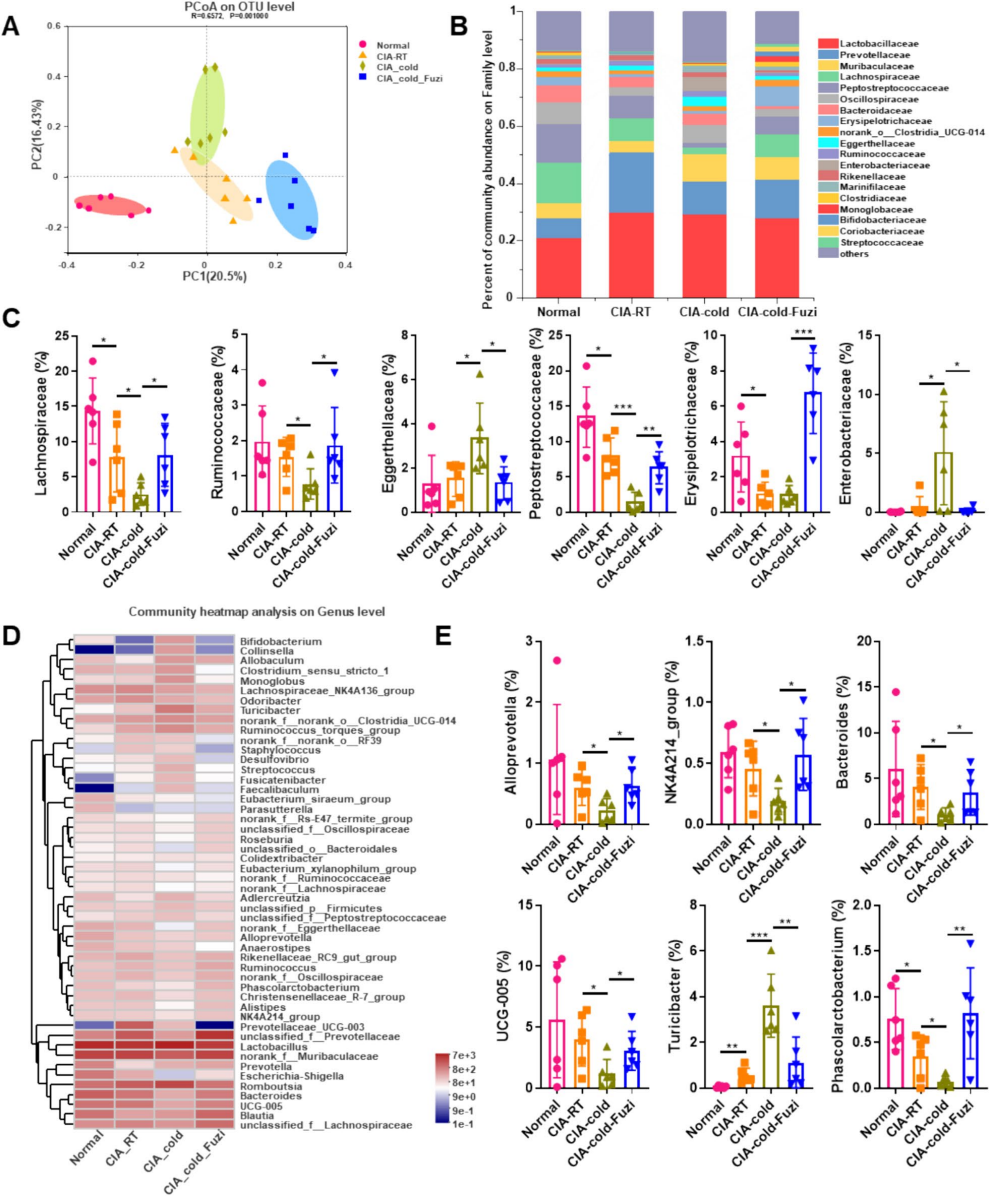
Regulatory effects of Fuzi on gut microbiota in CIA-cold rats. **A** Principal coordinates analysis (PCoA) plot of each rat's gut microbiota. **B** Percent taxa at the family level. **C** Relative abundance of taxa at the family level. **D** Heatmap of taxa at the genus level. **E** Relative abundance of taxa at the genus level

Since gut microbiota contributes to the development and progress of arthritis, we analyzed the effects of Fuzi on gut microbiota [18]. The *β*-diversity results evaluated by PCoA based on the weighted UniFrac distance matrix showed that the gut microbiota of the CIA-cold-Fuzi group was clearly separated from those of the Normal, CIA-RT, and CIA-cold groups, indicating that Fuzi had a significant impact on the gut microbiota composition (Fig. 3A). The results of the relative abundance of gut microbiota at the family level showed that compared to the CIA-cold group, the CIA-cold-Fuzi group had a significant increase in Lachnospiraceae, Ruminococcaceae, Peptostreptococcaceae, and Erysipelotrichaceae (*p* < 0.05), while Eggerthellaceae and Enterobacteriaceae were significantly reduced (*p* < 0.05) (Fig. 3B − C). At the genus level, the heatmap of the samples showed extensive changes in the clustering and composition of gut microbiota at the genus level induced by Fuzi treatment (Fig. 3D). Further analysis of the relative expression levels of the microbiota revealed that compared to the CIA-cold group, the CIA-cold-Fuzi group had significant increases in *Alloprevotella*, *NK4A214-group*, *Bacteroides*, *UCG-005*, and *Phascolarctobacterium*, while *Turicibacter* was significantly decreased (Fig. 3D, E). Fuzi significantly reversed some of the gut microbiota changes observed in the CIA-cold group from our previous study [11], including the family-level changes in Lachnospiraceae, Ruminococcaceae, and Eggerthellaceae, and the genus-level changes in *Alloprevotella*, *NK4A214-group*, and *Phascolarctobacterium*. In summary, Fuzi partially improved the dysregulated gut microbiota in CIA-cold rats.

**Fig. 4:**
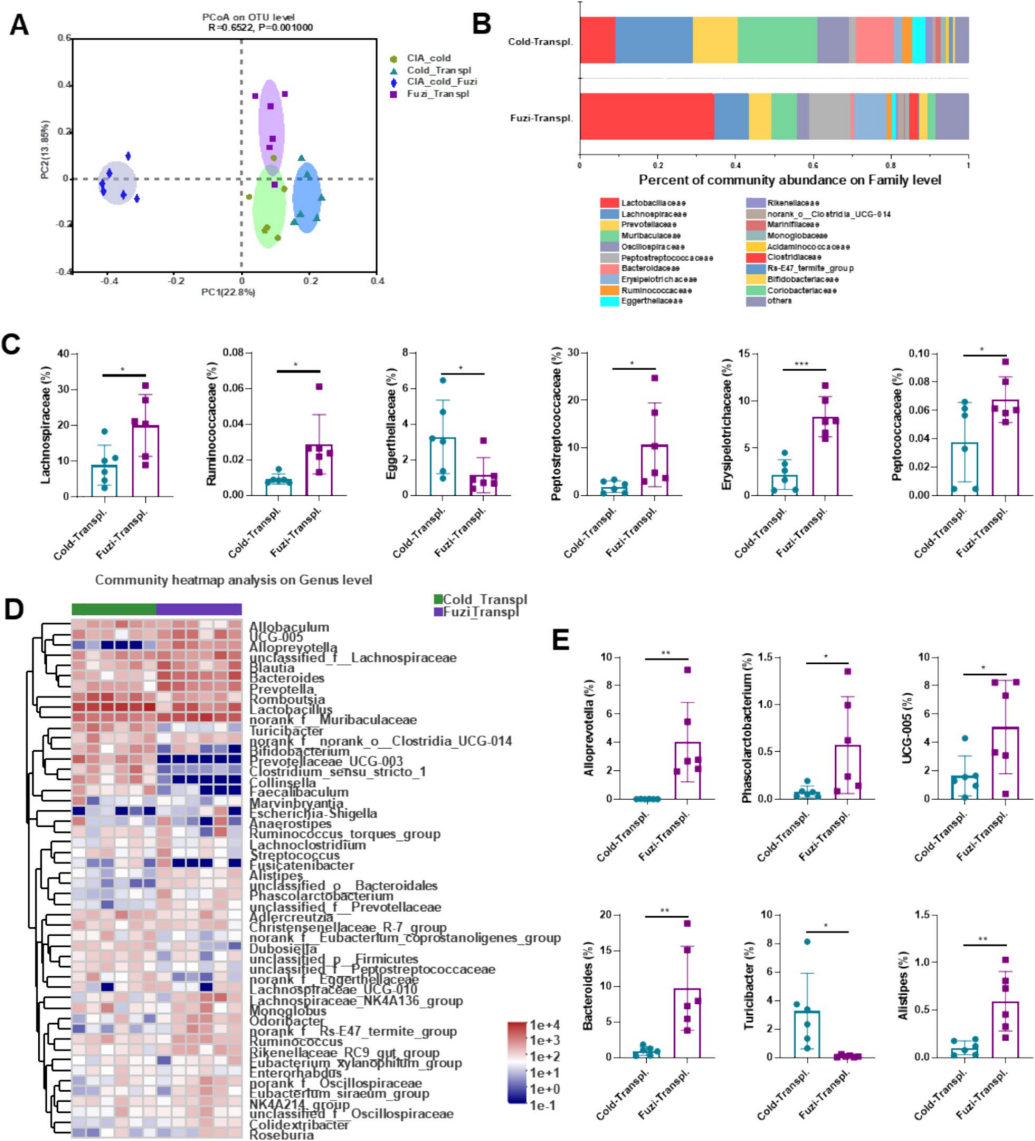
Effects of gut microbiota transplantation from the Fuzi-treated group on gut microbiota of CIA-cold rats. **A** PCoA plot of each rat's gut microbiota. **B** Percent composition of taxa at the family level. **C** Relative abundance of taxa at the family level. **D** Heatmap of taxa at the genus level. **E** Relative abundance of taxa at the genus level. Cold-Transpl.: cold-related collagen-induced arthritis receiving microbiota from CIA-cold group. Fuzi-Transpl.: cold-related collagen-induced arthritis receiving microbiota from CIA-cold-Fuzi group

To further confirm that the ameliorative effect of Fuzi on cold-related arthritis is dependent on gut microbiota, we transplanted the gut microbiota from CIA-cold rats and CIA-cold-Fuzi rats into CIA-cold rats (Fig. S1). 16S rRNA sequencing was performed to analyze the gut microbiota. PCoA showed that the gut microbial communities in the Fuzi-Transpl. group (recipients of gut microbiota from CIA-cold-Fuzi group) were similar to those in the CIA-cold-Fuzi group (donors of gut microbiota from Fuzi-treated CIA-cold rats). Likewise, the gut microbial communities in the CIA-cold group and the Cold-Transpl. group were also similar (Fig. 4A), indicating that the transplanted gut microbiota resembled their respective donors. Moreover, the trends of changes in Lachnospiraceae, Ruminococcaceae, Eggerthellaceae, Alloprevotella, UCG-005, and Phascolarctobacterium between the recipient groups (Cold-Transpl. group and Fuzi-Transpl. group) (Fig. 4B –E) were consistent with the trends observed between the donor groups (CIA-cold group and CIA-cold-Fuzi group) (Fig. 4B  –E). These changes in gut microbiota further demonstrate that the Fuzi-Transpl. rats (recipient rats) replicated the gut microbiota of the CIA-cold-Fuzi rats (donor rats).

**Fig. 5:**
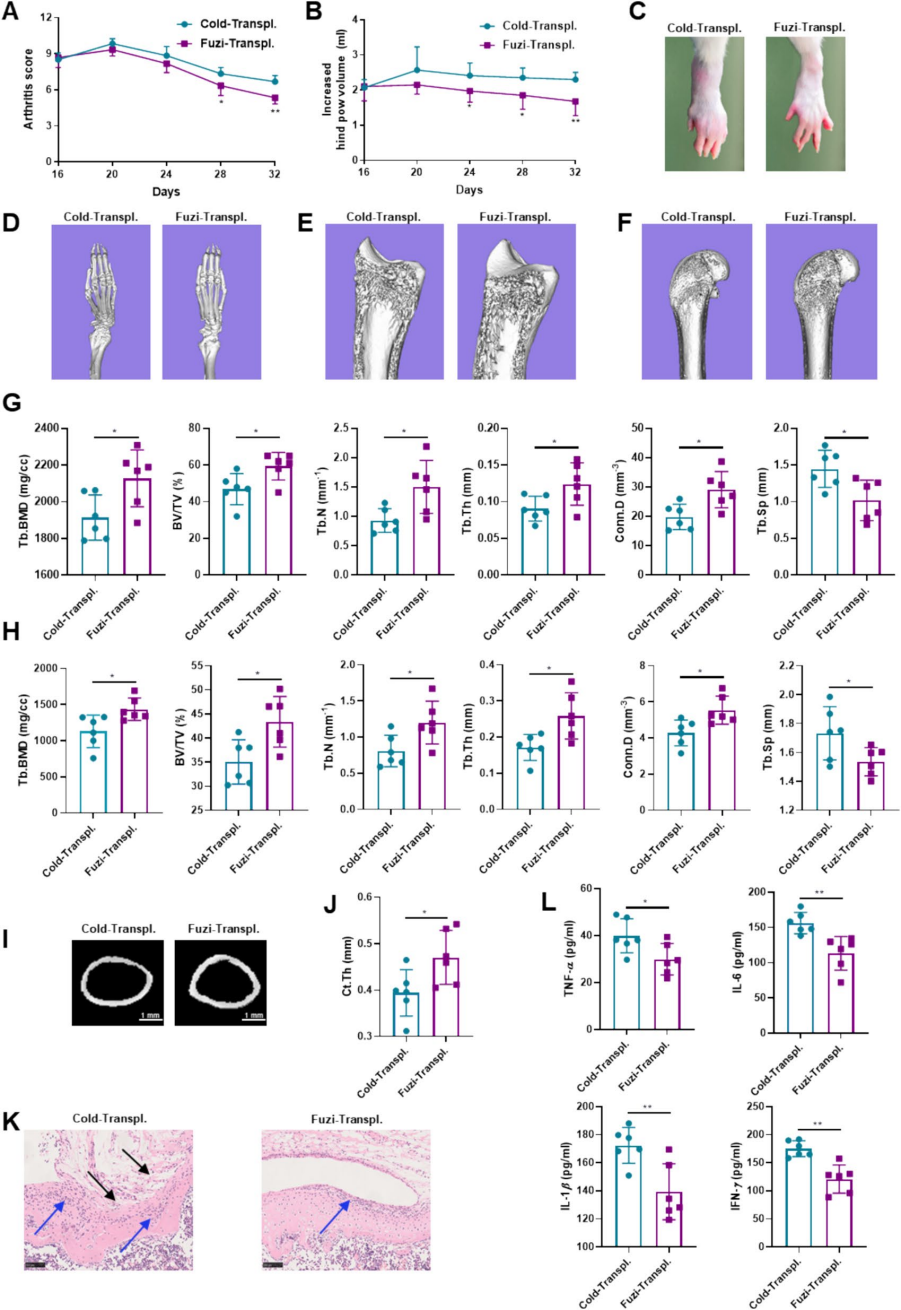
Effects of gut microbiota transplantation from the Fuzi-treated group on the severity of arthritis in CIA-cold rats. **A** Arthritis scores. **B** Paw swelling measurements. **C** Photos of the rat hind paws. **D−F** Micro-CT pictures of the rat ankle joint (**D**) proximal tibia (**E**) distal femur (**F**); **G** Trabecular bone parameters of the tibia. **H** Trabecular bone parameters of the femur. **I** Micro-CT pictures of the midshaft of the femur. **J** Cortical bone thickness (Ct.Th) of the midshaft femur. **K** H&E-staining of knee joints (× 200). **L** Serum levels of inflammatory cytokines, including TNF-*α*, IL-1*β*, IL-6, and IFN-*γ*

To further demonstrate that gut microbiota mediates the therapeutic effect of Fuzi on CIA-cold rats, we assessed the arthritis index, paw swelling, bone damage, knee joint inflammation, and serum inflammatory cytokines in Fuzi-Transpl. and Cold-Transpl. groups. Compared to the Cold-Transpl. group, the Fuzi-Transpl. group showed reduced arthritis index and paw swelling (Fig. 5A –C). Fuzi-Transpl. group also showed higher trabecular bone parameters of the tibia and femur, including BMD, BV/TV, Tb.N, Tb.Th, Conn.D, and Ct.Th of the femoral midshaft and higher Tb.Sp of the tibia and femur (Fig. 5D–J). In addition, compared with the Cold-Transpl. group, the cartilage loss (black arrows) and lymphocyte infiltration (blue arrows) in the knee joint tissue of Fuzi-Transpl. group were improved (Fig. 5K). Furthermore, Fuzi-Transpl. group showed significant lower levels of serum inflammatory cytokines (Fig. 5L) and significant higher levels of energy metabolism-associated hormones (Fig. S3A − F). Taken together, these results indicated that the ameliorative effect of Fuzi on cold-related arthritis is dependent on gut microbiota.

### Fuzi modulated microbial bile acid metabolism especially THDCA and TCA

**Fig. 6:**
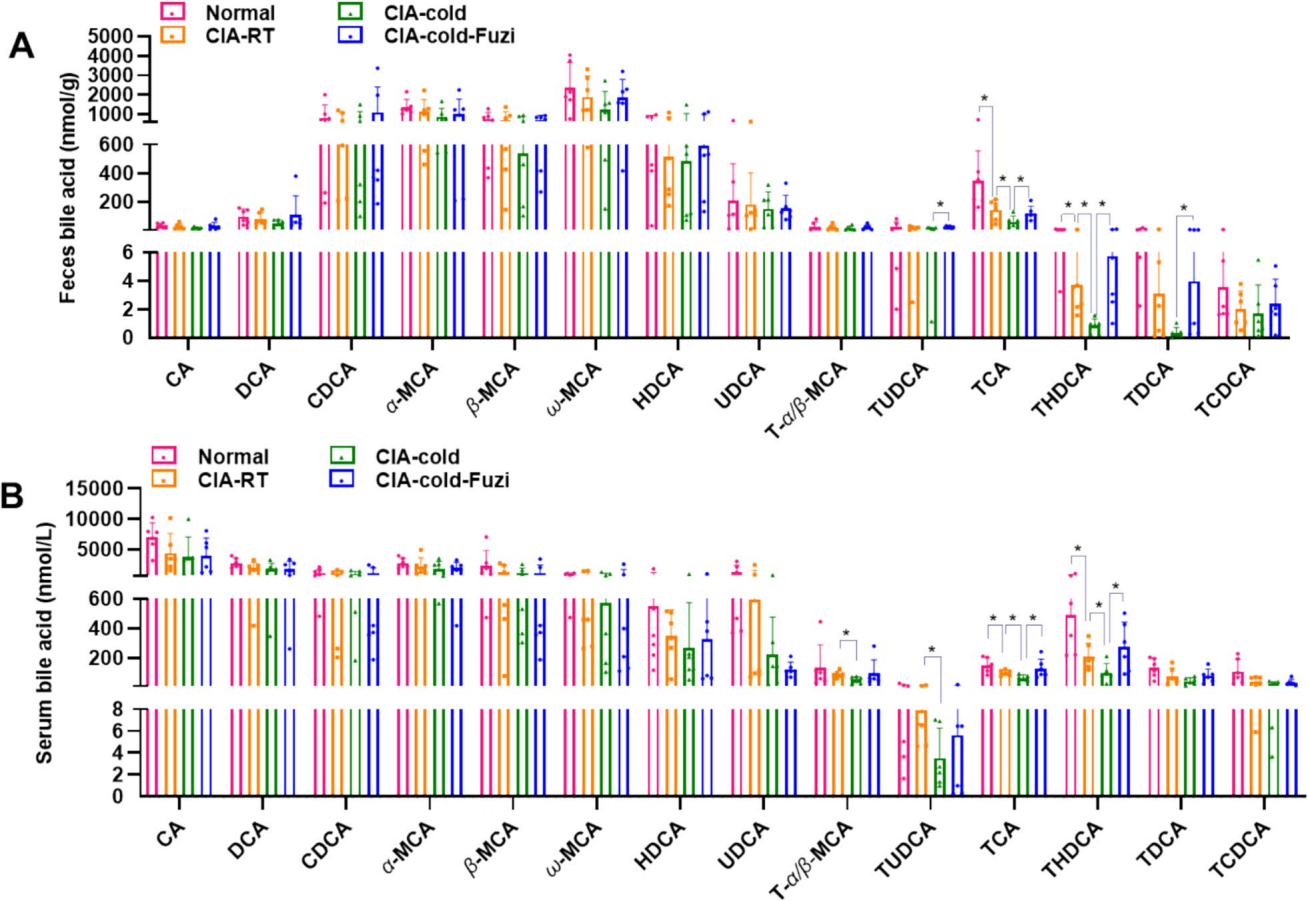
Effects of Fuzi on bile acid levels in CIA-cold rats. **A **,** B** Bile acid levels in feces (**A**) and serum (**B**)

It is reported that bile acids play important roles in inflammatory diseases and many herbal medicines can modulate the gut microbiota bile acid metabolism [19, 20]. We therefore first used targeted bile acid metabolomics to detect the effect of Fuzi on bile acid metabolism. In the feces, a total of 14 bile acids in all groups were detected (Fig. 6A). Except for TCA (a primary bile acid) and THDCA (a secondary bile acid), all the bile acids showed no significant content change between Normal group and CIA-RT group. In addition, the levels of TCA and THDCA in CIA-cold group were significantly lower than that of CIA-RT group. However, Fuzi treatment can significantly increase the levels of TCA and THDCA in cold exposed arthritis rats (Fig. 6A). To further confirm the change of bile acids, we also used targeted metabolomics to detect the levels of bile acids in serum. The levels of bile acids showed similar tendency of change in comparison with the levels of bile acids in feces (Fig. 6B). These results indicated that bile acids may contribute to the therapeutic effects of Fuzi on cold-related arthritis.

**Fig. 7:**
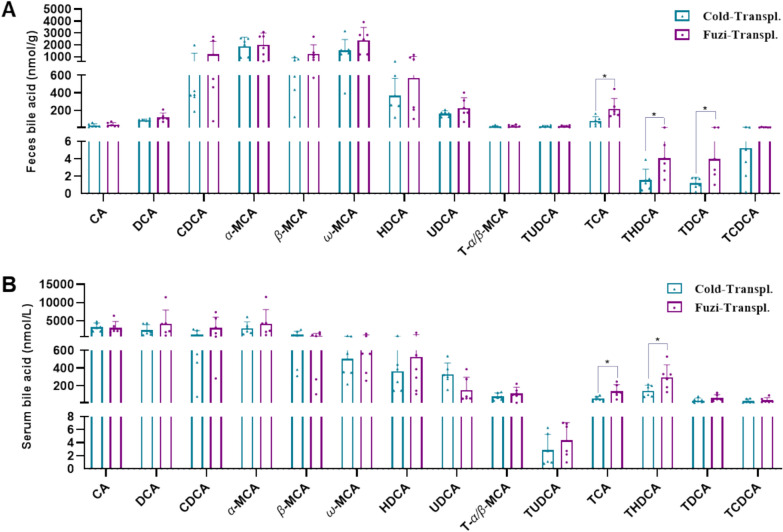
Effects of gut microbiota transplantation from the Fuzi-treated group on bile acid levels in CIA-cold rats. **A **,** B** Bile acid levels in feces (**A**) and serum (**B**)

To further confirm that bile acids are responsible for the therapeutic effects of Fuzi, we used targeted metabolomics to detect impact of FMT on the levels of bile acids. In the feces, the levels of TCA, THDCA, and TDCA in Cold-Transpl. group were significantly lower than that of Fuzi-Transpl. group, whereas the other bile acids showed no difference in these two groups (Fig. 7A). To further confirm the effects of FMT, we also used targeted metabolomics to detect the levels of bile acids in serum. Similar to the contents change of bile acids in feces, the levels of TCA and THDCA in Cold-Transpl. group were significantly lower than that of Fuzi-Transpl. group, whereas the other bile acids showed no difference in these two groups (Fig. 7A). These results suggested that TCA and THDCA may contribute to the therapeutic effects of Fuzi.

### TCA and THDCA show anti-inflammation effects on RAW264.7 cells

To explore the roles of TCA and THDCA in the treatment of arthritis, we conducted in vitro experiments to assess their anti-inflammatory activities. RAW264.7 cells, a macrophage-like cell line for studying inflammation in vitro, are widely employed in research on skeletal diseases such as arthritis and osteoporosis [21, 22]. Under the influence of inducers like lipopolysaccharide (LPS), RAW264.7 cells simulate an inflammatory response, releasing or upregulating various inflammatory mediators such as nitric oxide (NO), cyclooxygenase-2 (COX-2), TNF-α, and IL-6 [23]. We first examined the effects of different concentrations of TCA and THDCA on the viability of RAW264.7 cells using the CCK-8 assay to evaluate the cytotoxicity of TCA and THDCA. The results indicated that, compared to the control group, TCA and THDCA did not exhibit cytotoxicity within the concentration range of 1–5,000 μM after 24 h of treatment (Fig. S4A–B). TCA and THDCA significantly promoted cell proliferation within the concentration range of 10–200 μM, showing a dose-dependent effect between 10–100 μM, with the strongest proliferation observed at a concentration of 100 μM. However, at a concentration of 10,000 μM, TCA (p < 0.01) and THDCA (p < 0.001) significantly inhibited cell proliferation (Fig. S4A–B). Therefore, in subsequent experiments, we selected 10 μM, 50 μM, and 100 μM as the low, medium, and high concentrations of TCA and THDCA to study their anti-inflammatory effects in vitro.

**Fig. 8:**
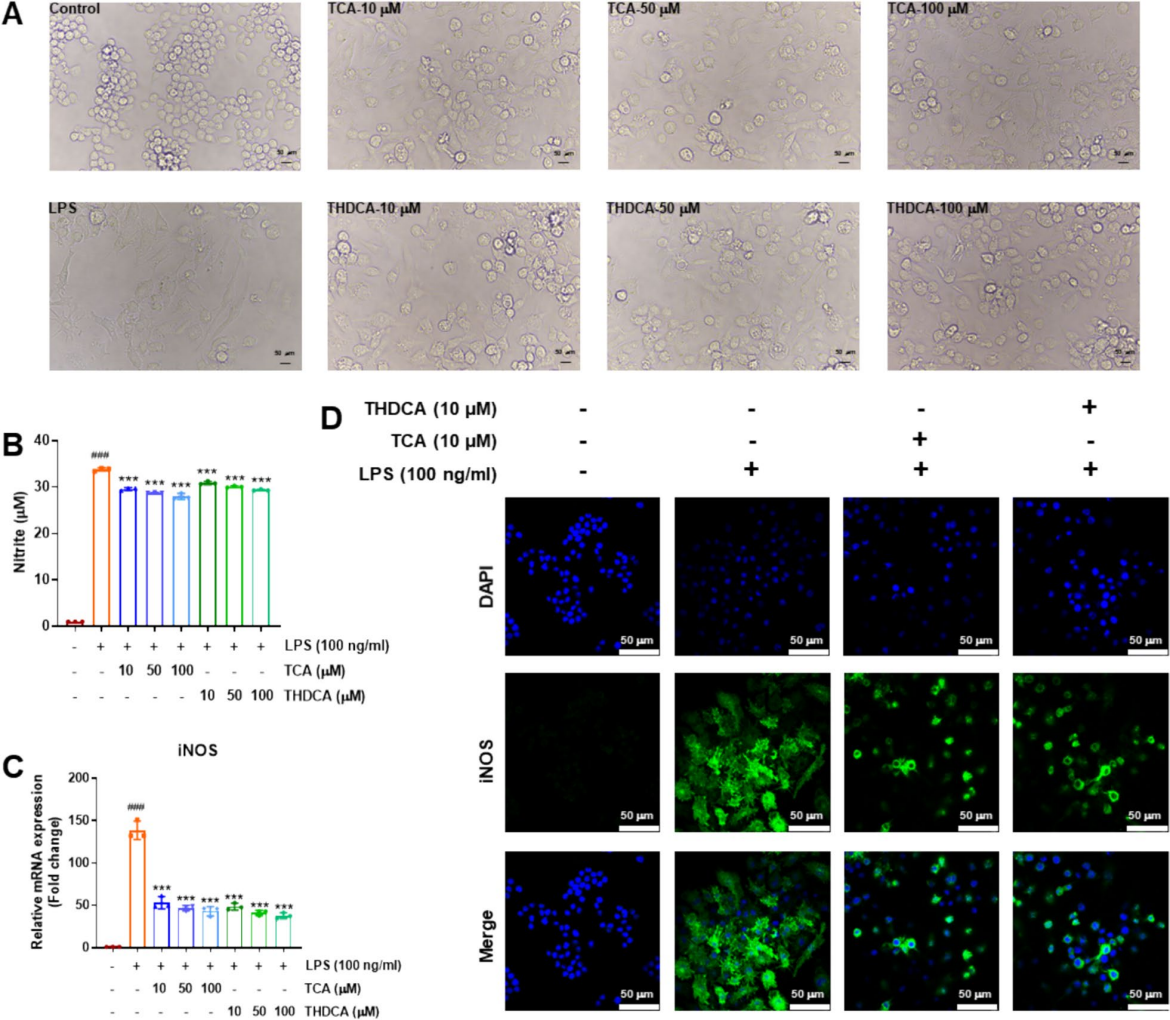
Effects of TCA and THDCA on RAW264.7 cell morphology, NO content, and iNOS expression. **A** Morphology of RAW264.7 cells (× 200). **B** Nitric oxide (NO) content. **C** iNOS mRNA expression levels. **D** Immunofluorescence staining for iNOS protein expression (× 600). ^###^*p* < 0.001 (compared with the control group) and ^***^p < 0.001 (compared with the LPS group)

LPS-induced morphological changes are indicative of RAW264.7 cell activation. To investigate the effects of TCA and THDCA on the activation of RAW264.7 cells, we analyzed the cell morphology using optical microscopy. RAW264.7 cells were cultured for 24 h under the influence of LPS, TCA, and THDCA. As shown in Fig. 8A, LPS stimulation for 24 h induced the extension and spreading of lamellipodia in RAW264.7 cells, while the normal group, which was not stimulated by LPS, did not exhibit any morphological changes. Treatment with TCA and THDCA for 24 h inhibited the LPS-induced morphological changes (Fig. 8A), indicating that TCA and THDCA suppressed the activation of LPS-stimulated RAW264.7 cells. Compared to the control group, NO secretion was significantly increased in the LPS-induced group. In the groups treated with TCA and THDCA, NO secretion was significantly lower than in the LPS-induced group and showed a dose-dependent effect (Fig. 8B). Next, we examined whether the reduction of NO in the TCA and THDCA groups was associated with decreased expression of iNOS. As expected, at the mRNA level, both TCA and THDCA significantly inhibited the LPS-induced increase in iNOS expression (Fig. 8C). Immunofluorescence staining further confirmed that TCA and THDCA reduced iNOS levels (Fig. 8D). In summary, these results indicate that TCA and THDCA suppressed LPS-induced activation of RAW264.7 cells, as well as inhibited LPS-induced NO production and iNOS expression.

**Fig. 9:**
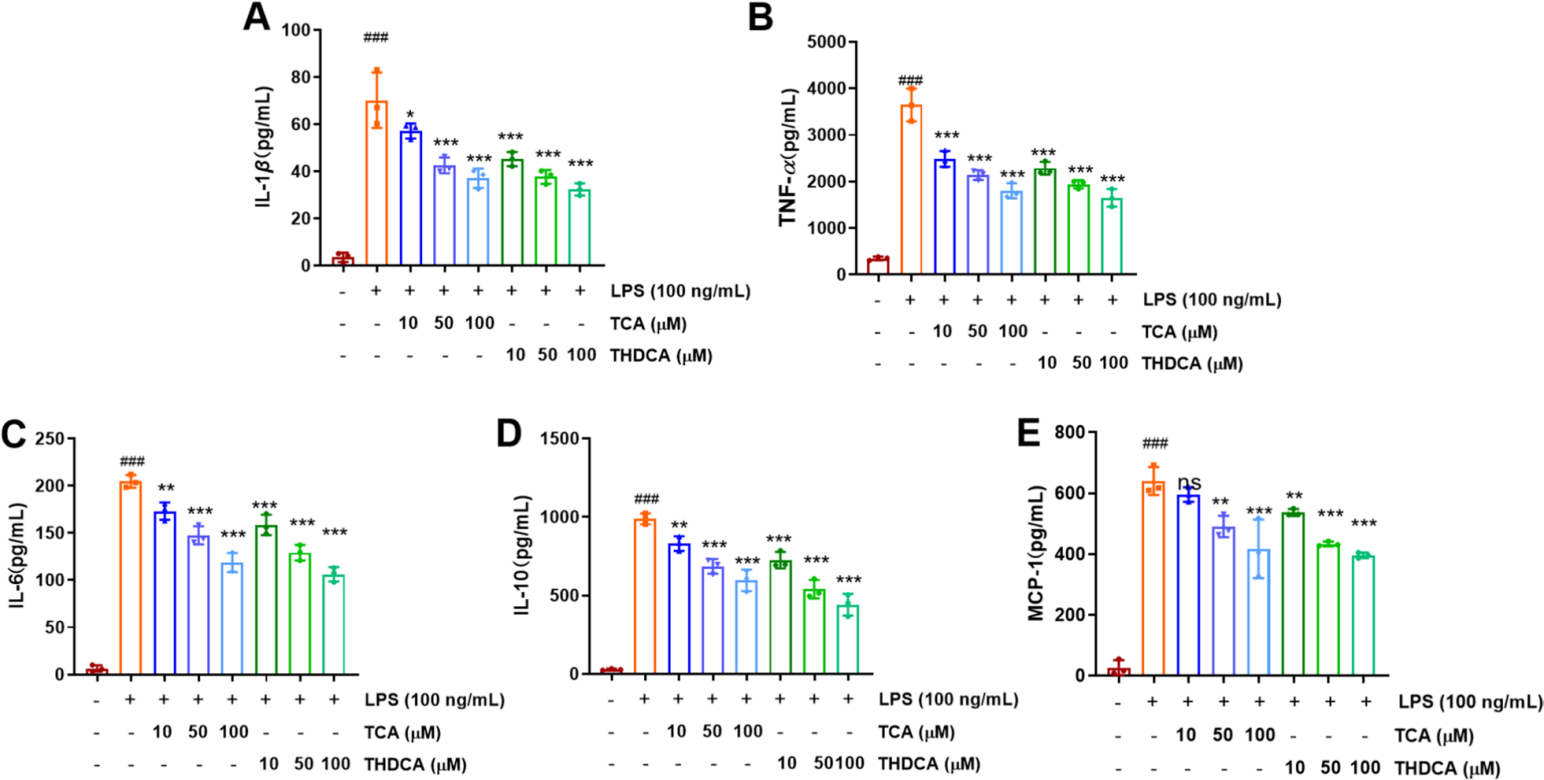
Effects of TCA and THDCA on cytokines in RAW264.7 cells. **A−E** The specific levels of IL-1*β* (**A**), TNF-*α* (**B**), IL-6 (**C**), IL-10 (**D**), and MCP-1 (**E**). ^###^*p* < 0.001 (compared with the blank control group); ^*^p < 0.05, ^**^p < 0.01, ^***^p < 0.001 (compared with the LPS group)

LPS stimulation of RAW264.7 cells induces the secretion of inflammatory cytokines, including IL-1*β*, TNF-*α*, IL-6, IL-10, monocyte chemoattractant protein-1 (MCP-1), and cyclooxygenase-2 (COX-2). To further investigate the effects of TCA and THDCA on LPS-induced inflammation in RAW264.7 cells in vitro, we measured the protein levels of IL-1*β*, TNF-*α*, IL-6, IL-10, MCP-1, and COX-2. The results showed that, compared to the control group, LPS stimulation of RAW264.7 cells increased the protein levels of IL-1*β*, TNF-*α*, IL-6, IL-10, MCP-1 (Fig. 9A–E, which was consistent with other reports [24]. Importantly, TCA and THDCA effectively inhibited the expression of these LPS-induced inflammatory cytokines, with even low doses of TCA and THDCA significantly reducing the relative protein levels of IL-1*β*, TNF-*α*, IL-6, IL-10, MCP-1, and COX-2 (Fig. 9A–E). These data suggest that TCA and THDCA have potential anti-inflammatory effects by suppressing the expression of inflammatory cytokines in RAW264.7 cells.

### Fuzi regulates TGR5-cAMP-PKA axis and NLRP3 inflammasome to reduce cold-related arthritis

**Fig. 10:**
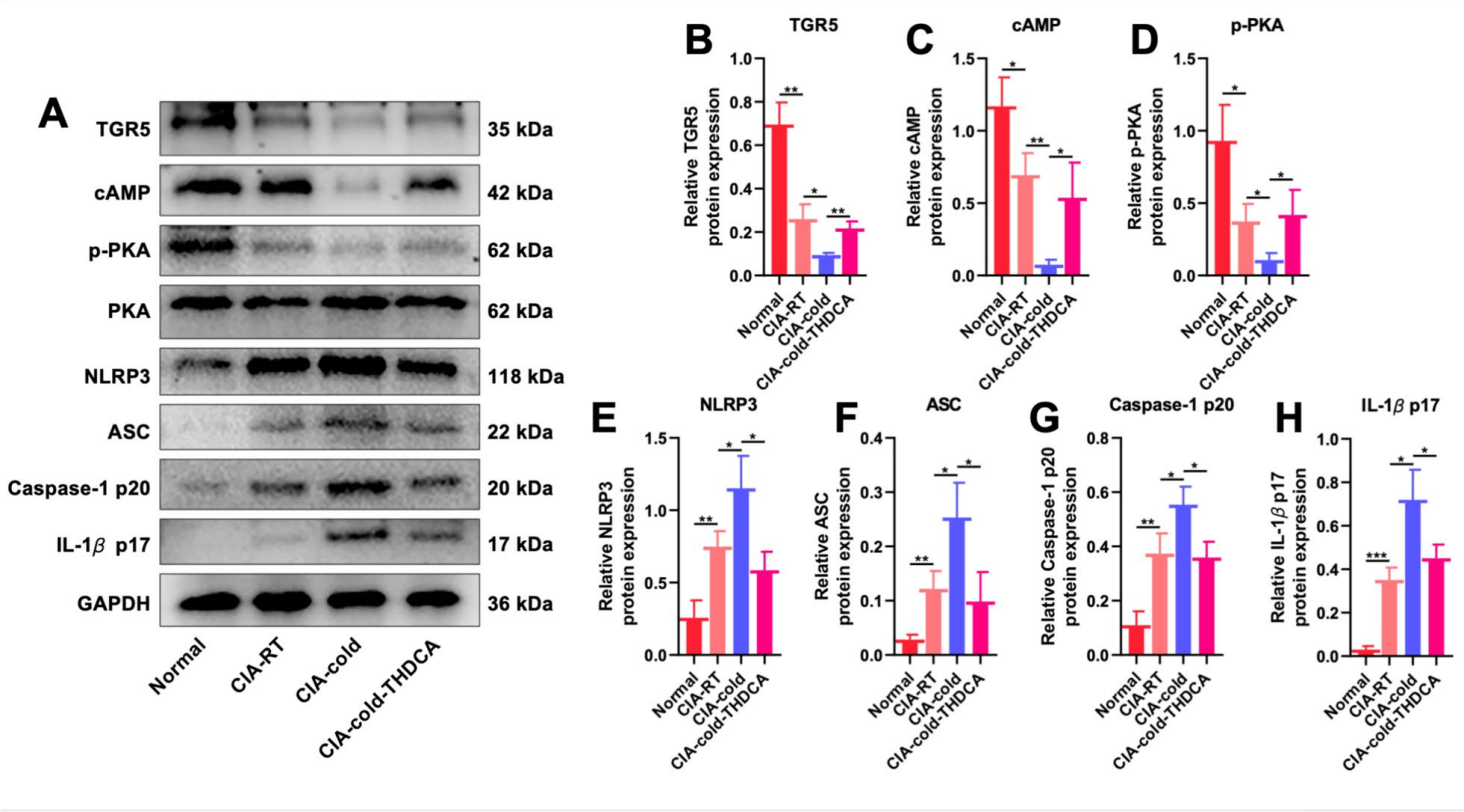
Fuzi inhibits NLRP3 inflammasome activation via the TGR5/cAMP/PKA signaling pathway. **A** Representative Western blotting bands. **B−H** Relative quantification of proteins TGR5 (**B**), cAMP (**C**), p-PKA (**D**), NLRP3 (**E**), ASC (**F**), Caspase-1 p20 (**G**), and IL-1*β* p17 (**H**)

Here we have demonstrated that Fuzi can regulate bile acid metabolism and increase both TCA and THDCA. In our previous study, we have demonstrated that THDCA, not TCA, improves arthritis induced by cold condition in CIA rats [11]. We therefore hypothesize that Fuzi can regulate the bile acid target and thus attenuate cold-related arthritis. To test our hypothesis, we used western blot to evaluate the levels of bile acid direct target Takeda G protein-coupled receptor 5 (TGR5) and the downstream signals regulated by TGR5. Not surprisingly, CIA-RT group showed lower level of TGR5 compared with normal group, and CIA-cold group showed lower levels of TGR5 compared with CIA-RT group (Fig. 10A, B), which was consistent with our previous study [11]. In addition, Fuzi treatment increased the level of TGR5 in cold-exposed CIA rats, suggesting that modulating bile acids plays an important role in treating cold-related arthritis. The direct downstream signals of TGR5, i.e. cyclic adenosine monophosphate (cAMP) and phospho-protein kinase A (p-PKA), also showed similar trend of change as the TGR5 (Fig. 10C, D). It is reported that NOD-like receptor thermal protein domain associated protein 3 (NLRP3) inflammasome triggered by TGR5-cAMP-PKA axis play a direct role in arthritis inflammation [11, 25], we therefore further determined the levels of NLRP3, ASC, caspase-1 p20, and IL-1*β* p17. The results showed that cold exposure can increase NLRP3, ASC, caspase-1 p20, and IL-1*β* p17 and Fuzi treatment can reduce the levels of these proteins in CIA-cold rats. Taken together, these results suggested that Fuzi regulates bile acids especially THDCA to modulate TGR5-cAMP-PKA signaling and NLRP3 inflammasome to reduce cold-related arthritis.

## Discussion

In TCM, RA can be generally classified into cold pattern and heat pattern [10], and cold exposure is an important environmental factor of cold pattern RA. Recent studies showed that cold exposure can modulate gut microbiota and microbial bile acid metabolism and thus affect the development of diseases such as hypertension, colitis, and obesity [26–28]. In our previous study, we demonstrated that cold exposure can aggravate arthritis via modulating gut microbiota-derived secondary bile acids and the downstream TGR5-cAMP-PKA signaling and NLRP3 inflammasome [11]. We have also previously demonstrated that Fuzi can modulate bile acids in both normal and cold environments [15, 17]. We therefore hypothesize that Fuzi can alleviate cold-related RA through modulating gut microbiota and bile acid metabolism, and the results confirmed our hypothesis.

In recent years, researchers have found that the gut microbiota is a factor shaping the development of RA [29]. In our study, the levels of taxa such as Lachnospiraceae and Ruminococcaceae were significantly elevated in CIA-cold-Fuzi group in comparison with CIA-cold group. These two taxa have been demonstrated to be linked to anti-inflammatory function in reducing inflammatory diseases like arthritis [30, 31]. Similar change can be seen in Eggerthellaceae, which is found to be a potential microbial taxa marker of CIA [32]. To test whether the alteration of the gut microbiota is correlated with or causal to the effects of Fuzi, we performed FMT and sequenced gut microbiota composition of recipient rats. Although antibiotics were not used in our study to remove the native gut microbiota in recipients, the results showed that microbiota transplantation retained the typical microbial signature of the donor rats, including Lachnospiraceae, Ruminococcaceae, Eggerthellaceae, etc. Furthermore, the recipient rats showed typical phenotype change of arthritis as their donors. Taken all these together, the results showed that Fuzi ameliorated cold-related arthritis through modulation of the gut microbiota.

Bile acids can be generally divided into primary bile acids and secondary bile acids. Primary bile acids are synthesized in the liver and discharged into the duodenum, whereas secondary bile acids are generated from primary bile acids by gut microbiota in the terminal ileum and colon [33]. Because Fuzi increased the levels of secondary bile acid producing taxa such as Lachnospiraceae and Ruminococcaceae [30, 34], we therefore used targeted metabolomics to investigate the effects of Fuzi on bile acids. The results showed that both primary bile acid TCA and secondary bile acids THDCA in feces and serum were increased after the treatment of Fuzi in CIA-cold rats, suggesting an important role of bile acid metabolism in the pharmacological effects of Fuzi. To further validate the role bile acid metabolism, we investigated the levels of bile acids in the feces and serum of the recipient rats including Fuzi-Transpl. and Cold-Transpl. rats. The results showed similar tendency to their respective donors, indicating the important roles of bile acids in achieving the pharmacological effects of Fuzi.

In our previous study, we investigated the effects of TCA and THDCA on CIA-cold rats [11]. The results showed that THDCA supplementation, but not TCA supplementation, ameliorated RA condition in CIA-cold rats. The reason may be linked to the fact that distinct bile acids, whether conjugated and unconjugated, possess divergent strengths in activation of their targets. It has been reported that activation of the NLRP3 inflammasome, caspase-1 and ASC, can contribute to the development of RA [35]. Bile acids show the ability to inhibit NLRP3 inflammasome activation via acting on TGR5-cAMP-PKA signaling to inhibit inflammatory diseases [36]. Since we found out that cold exposure can cause a decrease of THDCA, we evaluated the effects of Fuzi on the bile acid related TGR5-cAMP-PKA signaling and NLRP3 inflammasome. The results showed that Fuzi can act on TGR5-cAMP-PKA signaling and inhibit NLRP3 inflammasome, suggesting that Fuzi can inhibit cold-related arthritis via modulating gut microbial bile acid metabolism and subsequent activating of TGR5-cAMP-PKA signaling.

Disturbance of thyroid-related hormones such as T3 and T4 is a common feature of cold-related diseases [37]. It has been demonstrated that cold exposure can affect the energy metabolism of RA [38, 39]. In our study, Fuzi treatment increased the levels of thyroid-related hormones including T3, FT3, T4, FT4, TRH, and TSH. These hormones play important roles in energy metabolism by promoting adaptive thermogenesis in adipocytes, regulating cardiovascular functions such as heart rate, modulating glucose homeostasis, and affecting endogenous glucose production in the liver [40]. The results indicated that Fuzi increased the energy metabolism of rats with cold-related RA, which is in line with the hot property of Fuzi. In addition, the results also showed that Fuzi can act on the TGR5-cAMP-PKA signaling pathway, a pathway that can be activated by bile acids and promote energy metabolism by promoting T3 transformation in adipocyte [41]. Adipocytes are abundant in joint synovium. Therefore, one mechanism by which Fuzi promotes energy metabolism in arthritis joint maybe linked to regulating the TGR5-cAMP-PKA signaling pathway, which further promote T3 transformation in adipocytes and ultimately increase thermogenesis. However, further validation is needed to confirm this relationship.

Our study also has some limitations. Although FMT was used to confirm the role of gut microbiota, this evidence is not strong enough. Further studies using germ-free animals to confirm the causal role of gut microbiota in Fuzi-induced alleviation of cold-related RA are needed. In addition, we have screened possible key taxa such as Lachnospiraceae and Ruminococcaceae. Further studies using germ-free animals are needed to confirm their ameliorative effects on cold-related RA is need. Furthermore, fecal microbiota culture is needed to screen the specific bacterium responsible for production of HDCA. Lastly, the molecular relationship among THDCA, thyroid related hormones, and energy metabolism needs further study.

## Conclusion

Fuzi is a typical hot traditional Chinese medicine. In this study, measurements of the arthritis index, paw swelling, bone damage and joint inflammation indicated that Fuzi can alleviate cold-related RA. Gut microbiota composition analysis combined with FMT demonstrated that the ameliorative effect of Fuzi is dependent on gut microbiota. Targeted analysis of bile acids, in vitro analysis of the anti-inflammation effects of TCA and THDCA on RAW264.7 cells, and the Western blot showed that Fuzi-regulated bile acid metabolism can regulate TGR5-cAMP-PKA signaling and the NLRP3 inflammasome to reduce cold-related arthritis. Our study demonstrated that supplementation with Fuzi or THDCA can be of great importance for the prevention and treatment of cold-related RA.

## Supplementary Information

Supplementary Material 1.

## Data Availability

Data will be provided on reasonable request.

## Abbreviations

ASC: Apoptosis associated speck like protein containing a CARD
*α*-MCA: α-Muricholic acid
*β*-MCA: *β*-Muricholic acid
BMD: Bone mineral density
BV/TV: Bone volume fraction
CA: Cholic acid
cAMP: Cyclic adenosine monophosphate
Caspase-1: Cysteinyl-aspartate specific protease-1
CIA-cold: Cold-related collagen-induced arthritis
CIA-cold-Fuzi: Cold-related collagen-induced arthritis treated by Fuzi
CIA-cold-MTX: Cold-related collagen-induced arthritis treated by methotrexate
CIA-RT: Collagen-induced arthritis at room temperature
Cold-Transpl.: Cold-related collagen-induced arthritis receiving microbiota from CIA-cold
CDCA: Chenodeoxycholic acid
Conn.D: Connectivity density
COX-2: Cyclooxygenase-2
Cs.Th: Cross-sectional thickness
DCA: Deoxycholic acid
ELISA: Enzyme-linked immunosorbent assay
FMT: Fecal microbiota transplantation
Fuzi-Transpl.: Cold-related collagen-induced arthritis receiving microbiota from CIA-cold-Fuzi
GAPDH: Glyceraldehyde-3-phosphate dehydrogenase
H&E: Hematoxylin–eosin staining
HDCA: Hyodeoxycholic acid
IFN-*γ*: Interferon-*γ*
IL-10: Interleukin-10
IL-1*β*: Interleukin-1*β*
IL-6: Interleukin-6
LPS: Lipopolysaccharide
MCP-1: Monocyte chemoattractant protein-1
micro-CT: Microcomputed tomography
NLRP3: NOD-like receptor thermal protein domain associated protein 3
p-PKA: Phospho-protein kinase A
RA: Rheumatoid arthritis
T-*α*/*β*-MCA: Tauro-*α*/*β*-muricholate
Tb.N: Trabecular number
Tb.Sp: Trabecular separation
Tb.Th: Trabecular thickness
TCA: Taurocholic acid
TCDCA: Taurochenodeoxycholic acid
TDCA: Taurodeoxycholic acid
TGR5: Takeda G protein-coupled receptor 5
THDCA: Taurohyodeoxycholic acid
TNF-*α*: Tumor necrosis factor-*α*
TUDCA: Tauroursodeoxycholic acid
UDCA: Ursodeoxycholic acid
UPLC-MS/MS: Ultra-performance liquid chromatography coupled to a mass spectrometry

## References

[1] GBD 2021 Rheumatoid Arthritis Collaborators. Global, regional, and national burden of rheumatoid arthritis, 1990–2020, and projections to 2050: a systematic analysis of the Global Burden of Disease Study 2021. Lancet Rheumatol. 2023;5:e594-e610. 10.1016/S2665-9913(23)00211-4. 10.1016/S2665-9913(23)00211-4 PMC10546867 37795020

[2] Nilsson J, Andersson MLE, Hafström I, Svensson B, Forslind K, Ajeganova S, et al. Influence of age and sex on disease course and treatment in rheumatoid arthritis. Open Access Rheumatol. 2021;13:123–38. 10.2147/OARRR.S306378. 34079395 10.2147/OARRR.S306378 PMC8163636

[3] Gravallese EM, Firestein GS. Rheumatoid arthritis—common origins, divergent mechanisms. N Engl J Med. 2023;388:529–42. 10.1056/NEJMra2103726. 36780677 10.1056/NEJMra2103726

[4] Smolen JS, Aletaha D, McInnes IB. Rheumatoid arthritis. Lancet. 2016;388:2023–38. 10.1016/S0140-6736(16)30173-8. 27156434 10.1016/S0140-6736(16)30173-8

[5] Guo Q, Wang Y, Xu D, Nossent J, Pavlos NJ, Xu J. Rheumatoid arthritis: pathological mechanisms and modern pharmacologic therapies. Bone Res. 2018;6:15. 10.1038/s41413-018-0016-9. 29736302 10.1038/s41413-018-0016-9 PMC5920070

[6] Burmester GR, Pope JE. Novel treatment strategies in rheumatoid arthritis. Lancet. 2017;389:2338–48. 10.1016/S0140-6736(17)31491-5. 28612748 10.1016/S0140-6736(17)31491-5

[7] Smedslund G, Hagen KB. Does rain really cause pain? A systematic review of the associations between weather factors and severity of pain in people with rheumatoid arthritis. Eur J Pain. 2011;15:5–10. 20570193 10.1016/j.ejpain.2010.05.003

[8] Smedslund G, Mowinckel P, Heiberg T, Kvien TK, Hagen KB. Does the weather really matter? A cohort study of influences of weather and solar conditions on daily variations of joint pain in patients with rheumatoid arthritis. Arthritis Rheum. 2009;61:1243–7. 10.1002/art.24729. 19714599 10.1002/art.24729

[9] Lu C, Xiao C, Chen G, Jiang M, Zha Q, Yan X, et al. Cold and heat pattern of rheumatoid arthritis in traditional Chinese medicine: distinct molecular signatures identified by microarray expression profiles in CD4-positive T cell. Rheumatol Int. 2012;32:61–8. 10.1007/s00296-010-1546-7. 20658292 10.1007/s00296-010-1546-7 PMC3253282

[10] Wang W, Guan J, Li Z, Wang X. Rheumatoid arthritis characteristics and classification of heat and cold patterns - an observational study. Heliyon. 2023;9: e13439. 10.1016/j.heliyon.2023.e13439. 36873147 10.1016/j.heliyon.2023.e13439 PMC9975089

[11] Liu J, Peng F, Cheng H, Zhang D, Zhang Y, Wang L, et al. Chronic cold environment regulates rheumatoid arthritis through modulation of gut microbiota-derived bile acids. Sci Total Environ. 2023;903: 166837. 10.1016/j.scitotenv.2023.166837. 37689184 10.1016/j.scitotenv.2023.166837

[12] Liu J, Feng W, Peng C. A song of ice and fire: cold and hot properties of traditional Chinese medicines. Front Pharmacol. 2021;11: 598744. 10.3389/fphar.2020.598744. 33542688 10.3389/fphar.2020.598744 PMC7851091

[13] He G, Wang X, Liu W, Li Y, Shao Y, Liu W, et al. Chemical constituents, pharmacological effects, toxicology, processing and compatibility of Fuzi (lateral root of *Aconitum carmichaelii* Debx.): a review. J Ethnopharmacol. 2023;307: 116160. 10.1016/j.jep.2023.116160. 36773791 10.1016/j.jep.2023.116160

[14] Zhang Y, Zhou H, Liu J, Zhang D, Yue S, Peng C. Uncovering the mechanism of Fuzi and Baishao in treating rheumatoid arthritis using systems pharmacology and molecular docking. Future Integr Med. 2023;2:117–28.

[15] Liu J, Tan Y, Ao H, Feng W, Peng C. Aqueous extracts of Aconite promote thermogenesis in rats with hypothermia via regulating gut microbiota and bile acid metabolism. Chin Med. 2021;16:29. 10.1186/s13020-021-00437-y. 33741035 10.1186/s13020-021-00437-y PMC7980327

[16] Zhang D, Cheng H, Zhang Y, Zhou Y, Wu J, Liu J, et al. Ameliorative effect of Aconite aqueous extract on diarrhea is associated with modulation of the gut microbiota and bile acid metabolism. Front Pharmacol. 2023;14:1189971. 10.3389/fphar.2023.1189971. 37266146 10.3389/fphar.2023.1189971 PMC10229775

[17] Zhang D, Cheng H, Wu J, Zhou Y, Tang F, Liu J, et al. The energy metabolism-promoting effect of aconite is associated with gut microbiota and bile acid receptor TGR5-UCP1 signaling. Front Pharmacol. 2024;15:1392385. 10.3389/fphar.2024.1392385. 39323631 10.3389/fphar.2024.1392385 PMC11422068

[18] Lamba A, Taneja V. Gut microbiota as a sensor of autoimmune response and treatment for rheumatoid arthritis. Immunol Rev. 2024;325:90–106. 10.1111/imr.13359. 38867408 10.1111/imr.13359 PMC11338721

[19] Cai J, Sun L, Gonzalez FJ. Gut microbiota-derived bile acids in intestinal immunity, inflammation, and tumorigenesis. Cell Host Microbe. 2022;30:289–300. 10.1016/j.chom.2022.02.004. 35271802 10.1016/j.chom.2022.02.004 PMC8923532

[20] Cheng H, Liu J, Zhang D, Tan Y, Feng W, Peng C. Gut microbiota, bile acids, and nature compounds. Phytother Res. 2022;36:3102–19. 10.1002/ptr.7517. 35701855 10.1002/ptr.7517

[21] Zhou F, Mei J, Han X, Li H, Yang S, Wang M, et al. Kinsenoside attenuates osteoarthritis by repolarizing macrophages through inactivating NF-κB/MAPK signaling and protecting chondrocytes. Acta Pharm Sin B. 2019;9:973–85. 10.1016/j.apsb.2019.01.015. 31649847 10.1016/j.apsb.2019.01.015 PMC6804452

[22] Yan Y, Zhang LB, Ma R, Wang MN, He J, Wang PP, et al. Jolkinolide B ameliorates rheumatoid arthritis by regulating the JAK2/STAT3 signaling pathway. Phytomedicine. 2024;124: 155311. 10.1016/j.phymed.2023.155311. 38199156 10.1016/j.phymed.2023.155311

[23] Rhule A, Navarro S, Smith JR, Shepherd DM. Panax notoginseng attenuates LPS-induced pro-inflammatory mediators in RAW264.7 cells. J Ethnopharmacol. 2006;106:121–8. 10.1016/j.jep.2005.12.012. 16427227 10.1016/j.jep.2005.12.012

[24] Hwang SJ, Song YS, Lee HJ. Phaseolin attenuates lipopolysaccharide-induced inflammation in RAW 264.7 cells and zebrafish. Biomedicines. 2021;9:420. 10.3390/biomedicines9040420. 33924583 10.3390/biomedicines9040420 PMC8069760

[25] Gao J, Zhang H, Yang Y, Tao J. Therapeutic potential of targeting the NLRP3 inflammasome in rheumatoid arthritis. Inflammation. 2023;46:835–52. 10.1007/s10753-023-01795-5. 36897552 10.1007/s10753-023-01795-5

[26] Wang B, Liu J, Lei R, Xue B, Li Y, Tian X, et al. Cold exposure, gut microbiota, and hypertension: a mechanistic study. Sci Total Environ. 2022;833: 155199. 10.1016/j.scitotenv.2022.155199. 35417730 10.1016/j.scitotenv.2022.155199

[27] Sun L, Wang X, Zou Y, He Y, Liang C, Li J, et al. Cold stress induces colitis-like phenotypes in mice by altering gut microbiota and metabolites. Front Microbiol. 2023;14:1134246. 10.3389/fmicb.2023.1134246. 37113236 10.3389/fmicb.2023.1134246 PMC10126409

[28] Ziętak M, Kovatcheva-Datchary P, Markiewicz LH, Ståhlman M, Kozak LP, Bäckhed F. Altered microbiota contributes to reduced diet-induced obesity upon cold exposure. Cell Metab. 2016;23:1216–23. 10.1016/j.cmet.2016.05.001. 27304513 10.1016/j.cmet.2016.05.001 PMC4911343

[29] Zaiss MM, Joyce Wu HJ, Mauro D, Schett G, Ciccia F. The gut-joint axis in rheumatoid arthritis. Nat Rev Rheumatol. 2021;17:224–37. 10.1038/s41584-021-00585-3. 33674813 10.1038/s41584-021-00585-3

[30] Sinha SR, Haileselassie Y, Nguyen LP, Tropini C, Wang M, Becker LS, et al. Dysbiosis-induced secondary bile acid deficiency promotes intestinal inflammation. Cell Host Microbe. 2020;27:659-670.e5. 10.1016/j.chom.2020.01.021. 32101703 10.1016/j.chom.2020.01.021 PMC8172352

[31] Scher JU, Ubeda C, Artacho A, Attur M, Isaac S, Reddy SM, et al. Decreased bacterial diversity characterizes the altered gut microbiota in patients with psoriatic arthritis, resembling dysbiosis in inflammatory bowel disease. Arthritis Rheum. 2015;67:128–39. 10.1002/art.38892 PMC4280348 25319745

[32] Pu Y, Zhang Q, Tang Z, Lu C, Wu L, Zhong Y, et al. Fecal microbiota transplantation from patients with rheumatoid arthritis causes depression-like behaviors in mice through abnormal T cells activation. Transl Psychiatry. 2022;12:223. 35650202 10.1038/s41398-022-01993-z PMC9160267

[33] Liu J, Tan Y, Cheng H, Zhang D, Feng W, Peng C. Functions of gut microbiota metabolites, current status and future perspectives. Aging Dis. 2022;13:1106–26. 10.14336/AD.2022.0104. 35855347 10.14336/AD.2022.0104 PMC9286904

[34] Zeng H, Larson KJ, Cheng WH, Bukowski MR, Safratowich BD, Liu Z, et al. Advanced liver steatosis accompanies an increase in hepatic inflammation, colonic, secondary bile acids and Lactobacillaceae/Lachnospiraceae bacteria in C57BL/6 mice fed a high-fat diet. J Nutr Biochem. 2020;78: 108336. 10.1016/j.jnutbio.2019.108336. 32004929 10.1016/j.jnutbio.2019.108336

[35] Vande Walle L, Van Opdenbosch N, Jacques P, Fossoul A, Verheugen E, Vogel P, et al. Negative regulation of the NLRP3 inflammasome by A20 protects against arthritis. Nature. 2014;512:69–73. 25043000 10.1038/nature13322 PMC4126806

[36] Guo C, Xie S, Chi Z, Zhang J, Liu Y, Zhang L, et al. Bile acids control inflammation and metabolic disorder through inhibition of NLRP3 inflammasome. Immunity. 2016;45:802–16. 10.1016/j.immuni.2016.09.008. 27692610 10.1016/j.immuni.2016.09.008

[37] Roodaki M, Faridi P, Abolhasanzadeh Z, Karimzadeh I. Hot and cold: An old theory with modern applications. Trends Pharm Sci. 2018;4.

[38] Mao X, Li W, Chen W, Li Y, Wang Q, Wang X, et al. Exploring and characterizing a novel combination of paeoniflorin and talatizidine for the treatment of rheumatoid arthritis. Pharmacol Res. 2020;153: 104658. 10.1016/j.phrs.2020.104658. 32001347 10.1016/j.phrs.2020.104658

[39] Guo H, Niu X, Gu Y, Lu C, Xiao C, Yue K, et al. Differential amino acid, carbohydrate and lipid metabolism perpetuations involved in a subtype of rheumatoid arthritis with Chinese medicine cold pattern. Int J Mol Sci. 2016;17:1757. 10.3390/ijms17101757. 27775663 10.3390/ijms17101757 PMC5085781

[40] McAninch EA, Bianco AC. Thyroid hormone signaling in energy homeostasis and energy metabolism. Ann N Y Acad Sci. 2014;1311:77–87. 10.1111/nyas.12374. 24697152 10.1111/nyas.12374 PMC4451242

[41] Lun W, Yan Q, Guo X, Zhou M, Bai Y, He J, et al. Mechanism of action of the bile acid receptor TGR5 in obesity. Acta Pharm Sin B. 2024;14(2):468–91. 10.1016/j.apsb.2023.11.011. 38322325 10.1016/j.apsb.2023.11.011 PMC10840437

