# Supplementary material for: Fuzi alleviates cold-related rheumatoid arthritis via regulating gut microbiota and microbial bile acid metabolism
Source: Chin Med. 2025 May 15;20:64. doi: 10.1186/s13020-025-01123-z (PMC12079872; doi:10.1186/s13020-025-01123-z)
Supplement: Supplementary file 1 — Supplementary Material 1. [file 13020_2025_1123_MOESM1_ESM.docx]

**Fuzi alleviates cold-related rheumatoid arthritis via regulating gut microbiota and microbial bile acid metabolism**

**Juan Liu^a,b,c^,** **Dandan Zhang^a^, Yaochuan Zhou^a^, Jinlu Wu^a^, Wuwen Feng^a,c^**^*^**, Cheng Peng^a,c^**^*^

^a^ *State Key Laboratory of Southwestern Chinese Medicine Resources, School of Pharmacy, Chengdu University of Traditional Chinese Medicine, Chengdu 611137, China*

^b^ *TCM Regulating Metabolic Diseases Key Laboratory of Sichuan Province, Hospital of Chengdu University of Traditional Chinese Medicine, Chengdu 610032, China*

^c^ *Key Laboratory of the Ministry of Education for Standardization of Chinese Medicine, Chengdu University of Traditional Chinese Medicine, Chengdu 611137, China*

*Corresponding authors at: State Key Laboratory of Southwestern Chinese Medicine Resources, School of Pharmacy, Chengdu University of Traditional Chinese Medicine, Chengdu 611137, China; Key Laboratory of the Ministry of Education for Standardization of Chinese Medicine, Chengdu University of Traditional Chinese Medicine, Chengdu 611137, China (W. Feng and C. Peng).

E-mail addresses: jiaoxiake-1@foxmail.com (W. Feng), pengchengcxy@126.com (C. Peng).


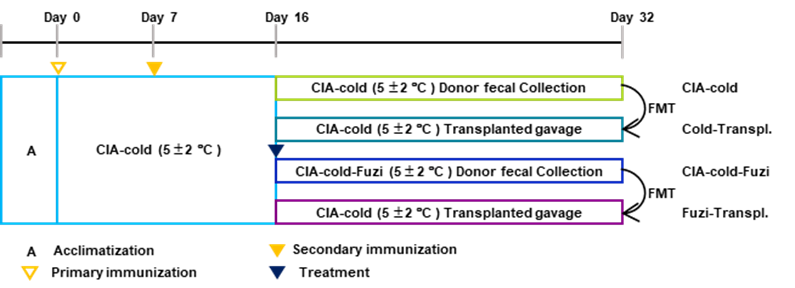
Figure S1. The flowchart for fecal microbiota transplantation.


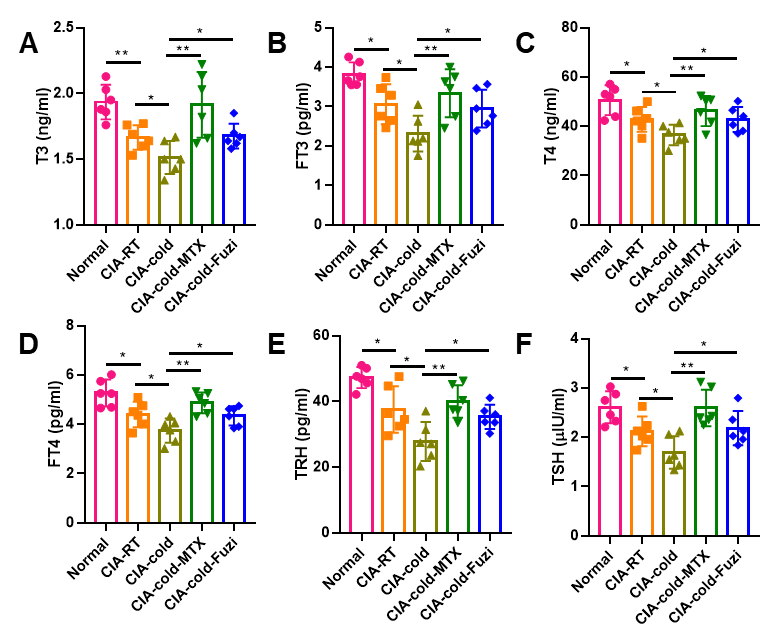


Figure S2. The effects of Fuzi on thyroid-related hormones.


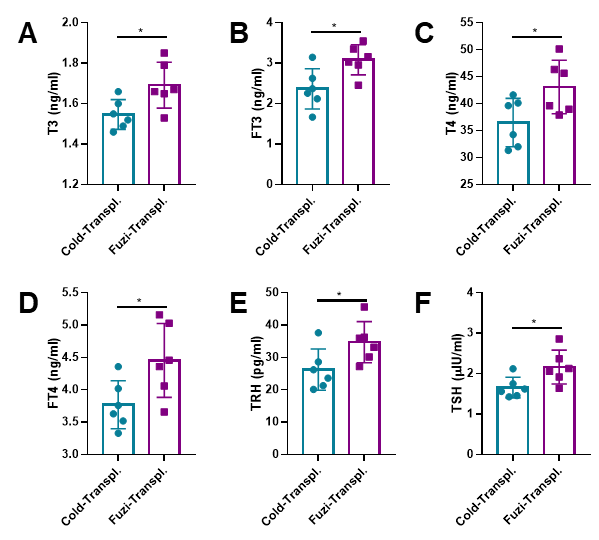


Figure S3. Effects of gut microbiota transplantation from the Fuzi-treated group on thyroid-related hormones in CIA-cold rats.


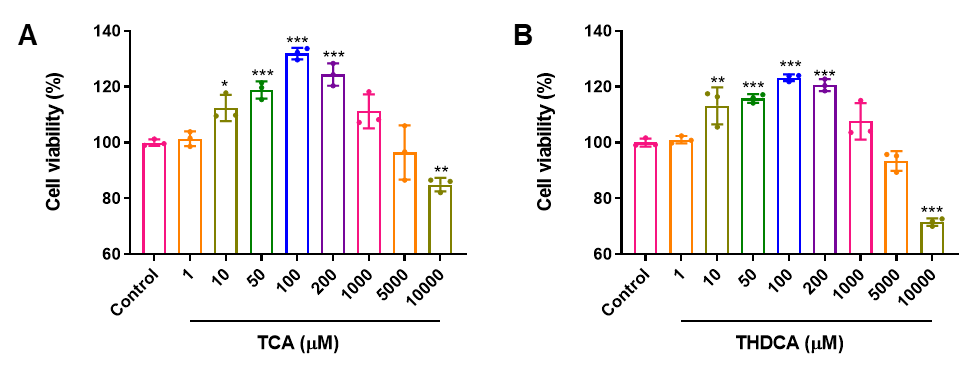
Figure S4. Determination of the TCA (A) and (THDCA) on cell viability at different concentration.
